# Flow Characteristics, Mechanical, Thermal, and Thermomechanical Properties, and 3D Printability of Biodegradable Polylactide Containing Boehmite at Different Loadings

**DOI:** 10.3390/polym13122019

**Published:** 2021-06-21

**Authors:** Dimakatso Makwakwa, Vincent Ojijo, Jayita Bandyopadhyay, Suprakas Sinha Ray

**Affiliations:** 1 DST-CSIR National Centre for Nanostructured Materials, Council for Scientific and Industrial Research, Pretoria 0001, South Africa; DMakwakwa@csir.co.za (D.M.); vojijo@csir.co.za (V.O.); jbandyopadhyay@csir.co.za (J.B.); 2 Department of Chemical Sciences, University of Johannesburg, Doornfontein, Johannesburg 2028, South Africa

**Keywords:** mechanical properties, biodegradable, thermomechanical properties, thermal properties, dicumyl peroxide, Joncryl

## Abstract

This work investigates the effects of modification of polylactide (PLA) using dicumyl peroxide (DCP) as a crosslinker and Joncryl as a chain extender on boehmite distribution. The PLA/boehmite (PLA/BA) composites at various concentrations were prepared via a twin-screw extruder. Transmission electron microscopy showed more agglomerations of BA particles when Joncryl and DCP were added individually to the PLA matrix, with lesser agglomeration upon simultaneous addition of DCP and Joncryl, which led to an enhancement of 10.7% of the heat distortion temperature and 8.8% of the modulus. The existence of fine dispersed BA particles in the BA3 sample improved the cold crystallization by 4 °C. Moreover, the maximum reinforcing effect in increasing the storage modulus of the prepared system was observed upon concurrent addition of DCP and Joncryl, with minimum reinforcing effect upon individual addition of DCP and Joncryl. In general, a bio-based PLA composite base BA with enhanced properties was successfully prepared for various applications.

## 1. Introduction

Interest in polylactide (PLA) has grown both industrially and academically due to its biocompatibility, biodegradability, and transparency [1]. Consequently, it has found application in the automotive, packaging, and medical industries, amongst others [2]. Regardless of its excellent properties, it also has limitations, such as poor thermal stability, brittleness, and slow crystallization during processing [3,4,5,6].

Generally, there are ways of improving the properties of PLA, including the addition of fillers or blending with other polymers [3,7,8,9,10]. For instance, Nieddu et al. [11] reported that 5 wt.% nanoclay increased the modulus of neat PLA. Other fillers, such as nano-silicon dioxide (SiO_2_) [12] and boehmite (BA) [10], have been found to improve the thermal stability, crystallizability, and mechanical and electrical conductivity of the polymers. Amongst these, BA has attracted more research interest due to its low cost, high flammability, high surface area, noble dispensability, and thermal stability [10,13]. Moreover, BA particles in nanometric form have greater reactivity than in micrometric form, owing to the increase of their surface area in nanometric dimensions [2]. A known challenge is the distribution of the hydrophilic BA in the hydrophobic PLA matrix, which leads to poor interfacial bonding between the polymer and the filler.

Several studies have been conducted on the modification of BA to improve the interfacial bonding between the polymer and the filler [10,13,14,15,16,17,18]. For instance, Malwela et al. [10] reported the impact of BA surface modification with *p*-toluene sulfonic acid on the mechanical, thermal, and rheological properties of PP/PS blends. Their results revealed that the addition of 1, 3, 5, and 7 wt.% modified BA particles in the PP/PS blend improved the modulus by 5.3, 13.5, 20.3, and 27.5%, respectively, due to the uniformly dispersed agglomerations of BA particles within the PS phase, whereas in the case of untreated BA, slight improvements of 0.2, 11.4, 17.8, and 25.7%, respectively, were observed, attributed to the agglomerations within the edges of the PS phase. On the other hand, the inclusion of 1 wt.% untreated BA particles had no effect on the crystallization temperature, while the crystallization temperature peaks moved from 116.90 to 119.0, 126.6, and 127.7 °C for the 3, 5 and 7 wt.%, respectively. Khumalo et al. [17] incorporated BA particles in low-density and high-density polyethylene (LDPE and HDPE). The authors revealed that 2 wt.% BA particles improved the resistance of the resulting composite to thermo-oxidative degradation from 0.5 to 2%. In 10 wt.% BA the improvement was from 1.5 to 3%, due to the presence of BA being reported for polyoxymethylene. Das et al. [2] reported the mechanical, thermal, and fire properties of biodegradable PLA/BA composites. Their results revealed that the incorporation of 3 wt.% BA nanoparticles increased the tensile strength of PLA by 57%, and the cold crystallization was observed in the range of 120–125 °C. However, this kind of modification includes a solvent, which has drawbacks, such as high cost and environmental problems.

One of the most common approaches to improving the distribution of the filler in the polymer matrix is through polymer modification [14,16]. Several studies on the interfacial modification of different polymers have been reported [1,19,20]. These modifications include Joncryl as a chain extender and DCP as an initiator. Joncryl is a polymeric chain extender with a low epoxy equivalent weight that reacts with the chain ends of polycondensates and effectively increases their melt viscosity. Additionally, it is a multifunctional oligomer chain extender that was intended to reverse the degradation in PLA; it has epoxy functional groups, which would react with the carboxyl and hydroxyl groups [1,20,21,22,23,24]. Joncryl has shown improvement in the interaction between carboxyl groups of PLA and the reactive functional epoxy group of the chain extender, and consequently, the enhancement of the crystallizability and mechanical properties of PLA [25]. Generally, DCP is a free radical generator in a polymer system, with the possibility of crosslinking or chain branching. Similarly, Joncryl helps with chain branching and extension in polyesters, such as PLA.

This study focuses on producing the PLA/BA composite in order to demonstrate the 3D printing of the samples. This study aimed to use the novel system to study the mechanical, flow, and thermal properties of PLA. We used DCP as a free radical generating agent in the reactive extrusion in order to enhance the amount of macroradicals that could introduce long-chain branching in the PLA chain. Joncryl, a patented, multifunctional, reactive polymer with improved thermal stability/chain extenders for specific food-contact applications, and polycondensation polymers including poly(ethylene terephthalate), was used as a crosslinking agent to extend the PLA chain and to improve the crystallizability of the PLA. Then, we demonstrated the 3D-orientability of the sample, with good distribution. This study aims to improve the BA particles’ distribution in the PLA matrix using chain extension and branching, which will lead to enhanced mechanical properties.

## 2. Materials and Methods

### 2.1. Materials

**Figure 1:**
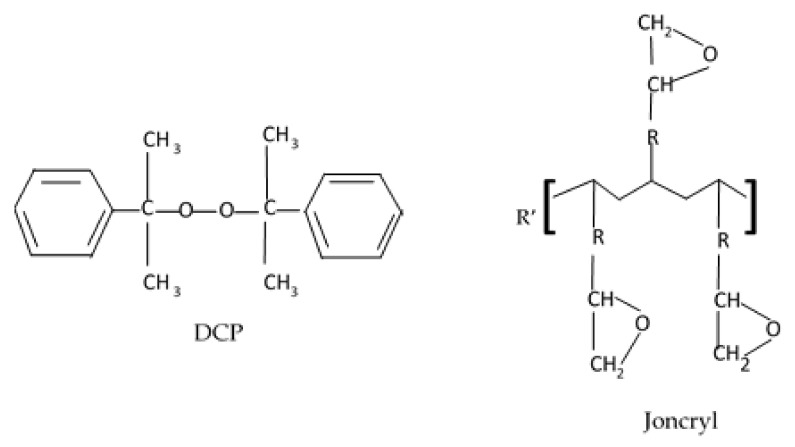
Chemical structure of dicumyl peroxide (DCP) and Joncryl.

The PLA used in this work was a commercial grade (PLA 4032D) purchased from NatureWorks LLC (Minnetonka, MN, USA), with a melt flow index (MFI) of 6 g/10 min (2.16 kg load) at 190 °C and a density of 1.23 g cm^−3^, while the BA powder was of a commercial grade manufactured by SASOL, under the trade name Dispersal 40, containing 80% Al_2_O_3_, donated by SASOL Germany. DCP was obtained from Sigma-Aldrich (Johannesburg, South Africa), with a molecular weight of 270.37 g/mol, density of 1056 g/mL, vapor pressure of 15.4 mmHg, and a melting point between 39 and 41 °C, the chain extender Joncryl ADR 4368 CS was donated by BASF South Africa. The chemical structures of DCP and Joncryl are shown in Figure 1.

### 2.2. Preparation of the Samples

Prior to processing, PLA was dried at 80 °C under vacuum for 12 h. The samples with different compositions (Table 1) were melt compounded in a co-rotating twin-screw extruder from Thermo Scientific, Waltham, MA, USA, with an L/D of 40. The extruder conditions were as follows: feeding rate 5.6 g/min; screw speed 202 rpm; and barrel temperatures from the hopper to the die were 140, 160, 180, 180, 180, 180, 180, 180, and 190 °C, respectively. The composites were then compression molded into different specimens using a Carver compression molder (Carver laboratory Model 973214A, Wabash, IN, USA) at a temperature of 190 °C and pressure of 1 MPa for 6 min, then cooled to room temperature.

### 2.3. Characterization

A Fourier-transform infrared (FTIR) spectroscope from Perkin Elmer (Model: Spectrum 100, Branford, CT, USA) was used to verify the chemical interactions between PLA, DCP, Joncryl, and BA within the wavelength range of 500–4000 cm^−1^. For all of the spectra, 32 scans were collected, with a resolution of 4 cm^−1^.

The compression-molded disc specimens were used for XRD measurements using an X’pert PRO diffractometer from PANalytical (EA Almelo, The Netherlands). The operating voltage was 45 KV, and the current was 40 mA. The exposure time and the scanning rate used were 29 min 45 s and 0.011°/min, respectively.

The distribution of BA particles in the PLA matrix was investigated using transmission electron microscopy (TEM) (JOEL, JEM 2100, Tokyo, Japan) with an acceleration voltage of 200 kV. The samples were prepared using a Leica (Austria) EM FC6 cryo-ultramicrotome at −100 °C, a cutting speed of 3 mm, and a feed rate of 80 nm. The samples were sliced using a diamond knife.

Differential scanning calorimetry (DSC) measurements were studied using a DSC-Q2000 instrument from TA Instruments, New Castle, DE, USA. Pellets with a mass of approximately 4–5 mg were heated from −20 °C to 190 °C at a rate of 10 °C/min, and then maintained at that temperature for 5 min. Samples were cooled to −20 °C at a rate of 10 °C/min and kept constant for 5 min, then heated to 190 °C at a rate of 10 °C/min. The heating and cooling cycles were conducted under nitrogen as the purge gas, with a flow rate of 25 mL/min for all samples. The glass transition temperature (*T_g_*), melting temperature (*T_m_*), enthalpy of fusion (Δ*H_m_*), crystallization temperature (*T_c_*), cold crystallization temperature (*T_cc_*), and enthalpy of cold crystallization (Δ*H_cc_*) were obtained.

The dynamic mechanical analysis (DMA) was conducted using a PerkinElmer DMA (Model 8000, Branford, CT, USA) analyzer in dual cantilever-bending mode. The temperature was measured at a frequency of 1 Hz, strain amplitude of 0.01%, and heating rate of 2 °C/min in the temperature range of −80 to +115 °C.

Heat distortion temperatures (HDTs) were measured using a CEAST HDT-VICAT instrument, and the measurements were recorded using the following conditions: Oil bath preheated to 30 °C; T_start_ = 30 °C; heating rate (ϕ) = 120 °C/h; T_max_ = 100 °C; end = 0.34 mm; span = 64 mm; and stress = 450 kPa.

The melt state rheological properties of neat PLA and composites were investigated using an Anton Paar stress/strain-controlled rheometer Physica MCR501 (Garz, Austria) with parallel plates of 25 mm in diameter. The injection-molded disc samples were used for this test. Frequency sweep tests were carried out from 0.1 to 100 rad/s. Each sample was melted in a parallel plate at 190 °C for 5 min to remove the remaining thermal history, and a dynamic strain sweep was then performed in order to determine the common linear region. The melt flow rate (MFR) properties of the neat PLA and composites were investigated using a melt flow meter (multiweight). The pelletized samples were used for this test, weighing 4 g per sample. 

Tensile tests were performed in order to determine the modulus, yield strength, and elongation at break of each material, using an Instron 5966 tester (Instron Engineering Corp., Norwood, MA USA) with a load cell of 10 kN, in accordance with ASTM 638D standards. The test was carried out under tension mode at a single strain rate of 5 mm/min at room temperature. The dog-bone-shaped specimens were analyzed.

## 3. Results

### 3.1. Flow Properties

The melt flow rates (MFRs) of the prepared samples are shown in Table 2. PLA shows a higher MFR, implying lower viscosity and ease of processability. The addition of DCP and Joncryl individually decreased the MFR of PLA by 32.3 and 50.2%, respectively. A decrease in the MFR of PLA/DCP could be attributed to chain–chain coupling, which results from the interaction of radicals generated by DCP on PLA chains, as shown in Supplementary Figure S1. On the other hand, Joncryl as a chain extender could have increased the molecular weight and viscosity of PLA due to the reaction between the epoxide groups of Joncryl and the carboxylic groups of PLA (see Supplementary Figure S2) [25]. The FTIR spectroscopy shows the reactions between the chain extenders, and will be discussed in the FTIR section. The simultaneous addition of both Joncryl and DCP did not significantly affect the MFR of PLA/DCP/J compared to the PLA/J system. Upon the addition of BA at 2 and 3 wt.% to PLA/DCP/J, the MFR decreased slightly, by 12.7 and 8.5%, respectively, suggesting the immobilization of PLA chains by the nanoparticles. However, with further increase in BA particles, the MFR started to increase, probably due to the separation and weakening of PLA chains by the rigid BA particles.

**Figure 2:**
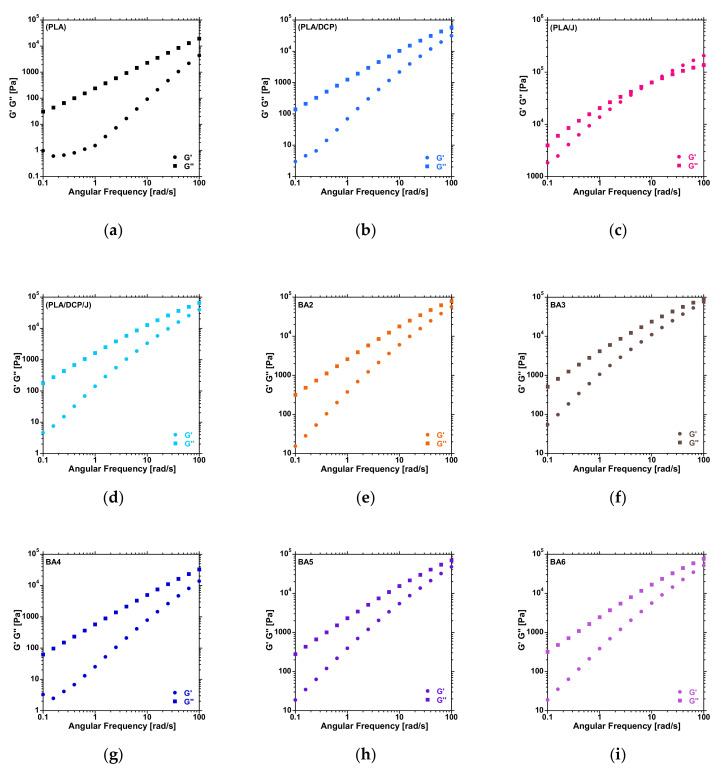
Storage and loss modulus curves of the PLA and BA composites at different contents.

DCP and Joncryl have a significant influence on the structural properties of polymers. In particular, the molecular weight (M_W_) and distribution (MWD) can be affected due to chain scission or chain–chain coupling in the presence of DCP. In addition, chain extenders such as Joncryl can also modify the structural properties of a polymer. Rheology is a powerful tool to elucidate the changes in the molecular structures of polymers in melt states. Figure 2 shows the plots of G′ and G″ against angular frequency from 0.1 to 100 rad/s. In these plots, the crossover point between G′ and G″ provides information about the changes in the M_W_ and MWD. The horizontal shift of the crossover frequency (G_ω_) to lower values is related to an increase in M_W_. In contrast, the vertical shift of the crossover modulus (G_m_) indicates an increased broadening of the MWD. It is worth mentioning that the crossover point is determined within the tested range (0.1 to 100 rad/s). For neat PLA (Figure 2a), the crossover point could not be determined within the tested range. However, looking at the distance between G′ and G″ at 100 rad/s, it can be noticed that the G′ and G″ curves are closer, suggesting lower angular frequency for the PLA/DCP (Figure 2b) system compared to neat PLA.

Moreover, a dramatic decrease in G_ω_ was noticed in the PLA/J system (Figure 2c), indicating an increase in the M_W_ of PLA. A horizontal shift to higher values could be noticed in the PLA/DCP/J system (Figure 2d); this suggests that there was a reduction in the M_W_ of PLA, which could be attributed to chain scission when both DCP and Joncryl were added. The incorporation of BA did not significantly change the M_W_ of the PLA/DCP/J system (Figure 2e–i), as can be seen from the distance between G′ and G″ at 100 rad/s.

**Figure 3:**
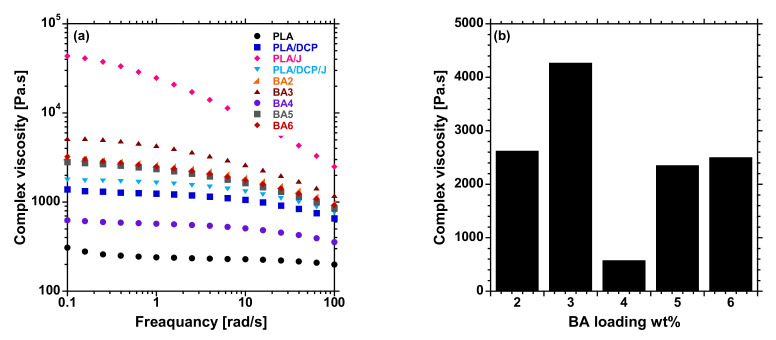
(**a**) Complex viscosity curves of neat PLA and BA composites at different loadings, and (**b**) viscosity at 1 frequency (rad/s) BA composites at different loadings.

Figure 3a shows the complex viscosity of the prepared PLA/DCP/J-based composites against angular frequency. As evident from Figure 3, the flow behavior of PLA, PLA/DCP, PLA/J, and PLA/DCP/J followed a similar trend to that noted from the MFR analysis. However, PLA/J showed the highest viscosity due to an increase in the M_W_ when Joncryl was introduced to PLA. This observation is attributable to the branches formed by the Joncryl and the introduction of long-chain branching (LCB) in the PLA structure, conveying pseudosolid-like behavior. The viscosity of the composites was solely dependent on the distribution of BA particles in the PLA matrix. The BA3 system showed better distribution, and exhibited a higher viscosity than the other composites (Figure 3b). With the increase in filler concentration, the viscosity decreased, possibly due to the poor distribution and agglomeration of BA particles, forming the weak points in the matrix. Further increase in BA concentrations resulted in an increase in viscosity, due to the reinforcing effect of the particles.

### 3.2. Chain Extension

**Figure 4:**
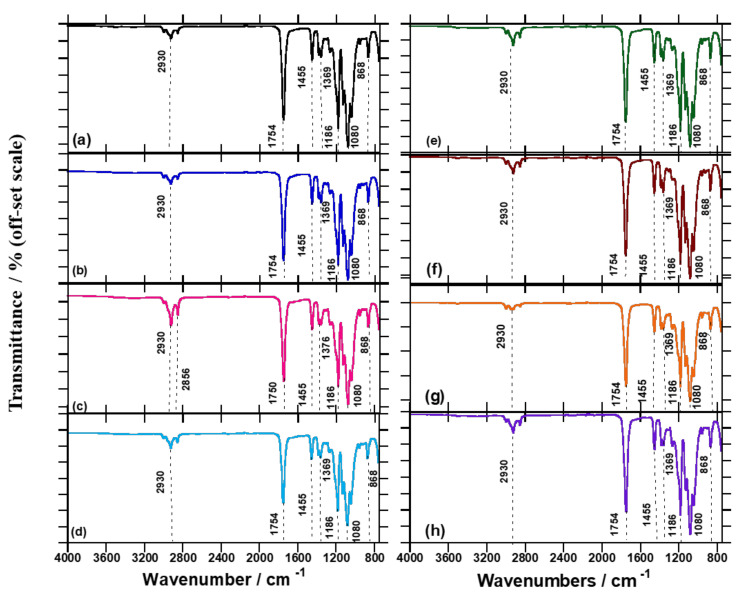
FTIR spectra for (**a**) neat PLA, (**b**) PLA/DCP, (**c**) PLA/J, (**d**) PLA/DCP/J, (**e**) PLA/BA, (**f**) PLA/BA/DCP, (**g**) PLA/BA, and (**h**) PLA/BA/DCP/J samples.

FTIR spectroscopy was used to compare the processed PLA/BA chain extender and crosslinking systems, in order to identify the reaction that may have transpired between the PLA/BA and chain extender/crosslinking. Figure 4a shows the FTIR spectra of neat PLA and composites. In the case of neat PLA, the peaks at 1754, 1455, and 1369 cm^−1^ are due to C=O stretching, C–H deformation, and C–O–H bands, respectively. Meanwhile, 1186 and 1080 cm^−1^ are assigned to –C–O stretching, 868 cm^−1^ is attributed to –C–C stretching, and 755 cm^−1^ is attributed to C–H bending. The H–O–H peak at 1630 cm^−1^ was not obtainable from the processed PLA, due to the existence of thermal chain scission at the C–O bond [26]. Supplementary Figure S3 shows the vibrations at 2852 and 3000 cm^−1^ assigned to the O–H stretching, and 2922 cm^−1^ due to the axial C–H stretching bond [27]. The FTIR spectra of neat BA (Supplementary Figure S4a) reveal that the vibrations at 3309 and 3095 cm^−1^ relate to the O–H stretching of BA [28]. The vibration at 1397 cm^−1^ is attributed to the amorphous surface structure that exists in crystalline BA [29]. In the case of neat DCP, as shown in Supplementary Figure S4b, vibrations are observed at 1727 cm^−1^ due to the C=O stretching, at 910 cm^−1^ due to C=C stretching, and at 762 cm^−1^ and 698 cm^−1^ due to C–H bending. Supplementary Figure S4c shows that in the FTIR spectra for neat Joncryl, vibrations are observed at 907 and 843 cm^−1^, attributed to the symmetric and asymmetric ring deformation of cyclic epoxide [30,31].

Figure 4e shows that there is no significant change to the PLA matrix upon addition of BA, due to the low concentration of BA embedded in the PLA matrix. Further, upon addition of DCP to the PLA/BA composite, as shown in Figure 4f, DCP undergoes homolytic cleavage when heat is applied, breaking down into free radicals and assisting in the removal of H’s from the PLA chains in order to create free radicals on the backbone structure of PLA, as shown in Supplementary Figure S1. This phenomenon is attributed to the propagation of the radical reaction to form a crosslinked/branched structure of PLA [13]. Therefore, PLA produces free radicals on the tertiary C atoms, which become stabilized in reactive extrusion [4]. In the case of Joncryl, the 907 and 843 cm^−1^ peaks disappear, owing to the interaction of the epoxy groups with carboxyl groups on the PLA, suggesting that the reaction occurrs between the Joncryl epoxy and the PLA terminal functional group [32]. Surprisingly, there is a synergistic effect of peak decreases for Joncryl at 2922 cm^−1^. These results relate to the decrease observed in the MFR results and the peak decrease shown in Supplementary Figure S2. Upon addition of all the components, the peak at 2922 cm^−1^ increases, suggesting that the initiator has created macroradicals after the chain extender has truly extended the PLA chain for the BA attachment. In all of the composites, vibrations at 1752, 1455, 1186, 1080, and 868 cm^−1^ remain unchanged. Inata and Matsumura [26] reported that the epoxides might react with carboxyl and hydroxyl end groups of polyesters, and the electrophilic group with the carboxyl end groups. It can be concluded that the chain extension/crosslinked/branched structures in the polymer composites play an important role in improving the properties of the reactive composites in a controlled way.

### 3.3. BA Distribution

**Figure 5:**
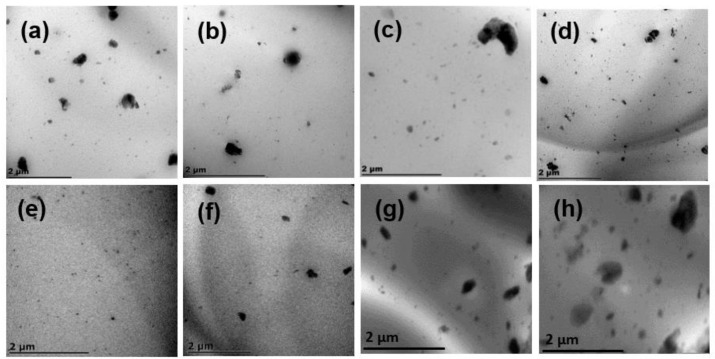
TEM images of (**a**) PLA/BA, (**b**) PLA/DCP/BA2, (**c**) PLA/J/BA2, (**d**) PLA/DCP/J/BA2, (**e**) BA3, (**f**) BA4, (**g**) B5, and (**h**) BA6.

To measure the distribution of BA particles in the PLA matrix, samples containing different BA concentrations were cryosectioned and viewed under TEM. Figure 5a illustrates that the particles of BA formed more agglomerates in the PLA matrix, and that the particles were not well distributed. Figure 5b shows that the addition of the initiator in the PLA matrix decreased the agglomerations of BA particles and distributed the particles better than in PLA/BA. This is attributed to the fact that the viscosity of the PLA matrix was increased by chain extension and/or branching, which assisted in breaking the BA agglomerates. On the other hand, better distribution of BA particles was observed in the presence of Joncryl, due to more chain branching that was created in the PLA structure. Furthermore, Figure 5d shows that the addition of all components at once produced a fair distribution of BA particles and strong intercomponent bonding. This observation correlates with the FTIR spectroscopy results in the next section, which show the interfacial bonding between all components.

Furthermore, it is evident from Figure 5e that upon the addition of 3 wt.% BA to the PLA matrix, the finest distribution was observed, showing an optimal distribution amongst all composites, owing to better intercomponent bonding amongst the neat PLA and BA particles. Das et al. [2] reported similar results in PLA/BA, revealing that the 3 wt.% BA loading in the PLA matrix was the optimal distribution. This led to improved mechanical properties, which were affected by the distribution of the BA particles in the PLA composites. On the other hand, the increase in BA concentration (i.e., 4, 5, 6, 10, and 20 wt.%) produced poorer distribution and more agglomerations of the BA particles in the PLA matrix.

### 3.4. Non-Isothermal Crystallization of the Modified PLA Systems

**Figure 6:**
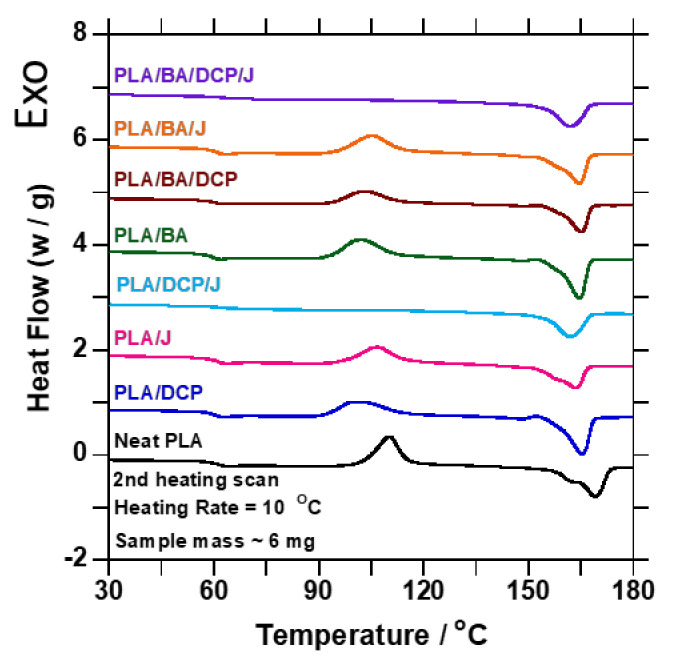
DSC traces of the second heating curve of neat PLA and samples containing DCP/Joncryl and BA.

DSC was used to study the effects of BA, DCP, Joncryl, and the resultant structures on the crystallization and melting temperature of the PLA matrix. The DSC data are summarized in Table 3. The degree of crystallinity (*χ_m_*) during cold crystallization, during heating (*χ_cc_*), and total crystallinity (*χ_c_*) were calculated using the following equations [2,23]:

(1) $$X_{m}=\frac{\Delta H_{m}}{\begin{array}{cc} \emptyset_{PLA} & \Delta H_{m}^{{}^{\circ}} \end{array}}$$

or

$$X_{cc}=\frac{\Delta H_{cc}}{\begin{array}{cc} \emptyset_{PLA} & \Delta H_{m}^{{}^{\circ}} \end{array}}$$

(2) $$X_{c}=X_{m}-X_{cc}$$

where Δ*H_m_* is the melting enthalpy, Δ*H_cc_* is the enthalpy of cold crystallization, *∅PLA* is the weight fraction of PLA, and Δ*H°_m_* is the enthalpy of fusion of 100% PLA, taken as 93.7 J/g [33]. Figure 6 shows the DSC thermograms from the second heating. PLA shows diverse transitions; the first transition is related to the PLA *T_g_* at (60 °C), the second transition is allied with the *T_cc_* at (110.24 °C), and the last transition is linked with the *T_m_* at (169.11 °C). In addition, Figure 6a also shows various melting temperature peaks at 164.13 and 169.11 °C. Moreover, there was no *T_c_* detected for PLA during the cooling cycle, because PLA crystallizes very slowly [34]. Upon addition of BA, and chain extension/branching by DCP/Joncryl, the *T_m_* of the samples moved to the low side of the graph compared to neat PLA. This observation suggests that BA acted as a weak nucleating agent in the samples; meanwhile, 3 wt.% showed a profound shift to the lower side of the *T_m_* peak (165.5 °C), suggesting a strong nucleating effect. Similar nucleating effects were reported by Malwela et al. [10] and Das et al. [2]. On the other hand, the *T_cc_* temperatures were also affected by the addition of BA, due to the nucleating effect. Upon addition of BA, the *T_c_* value of the composites decreased, moving towards the lower crystallization temperatures, indicating enhanced nucleation. Malwela et al. [10] reported a similar nucleating effect.

Additionally, BA limited the mobility of the PLA macromolecules, restricting their chain arrangement. When the molecular structure of PLA was altered by DCP and Joncryl, the *T_cc_* was reduced, and the crystallinity increased. This phenomenon is related to PLA degradation [35,36,37]. However, when the BA content was increased, the crystallinity in 3 wt.% decreased due to the well-dispersed BA particles causing a physical barrier in the PLA matrix. The *T_g_* of the samples remained unchanged regardless of incorporating BA or alterations to the molecular structure of PLA. This observation is related to the DMA results that will be discussed later. Overall, the *T_m_* and *T_c_* of the composites decreased compared to the neat PLA, confirming that BA, DCP, and Joncryl are good nucleating agents. Small loading of the nucleating agent assisted in forming the polymer crystals; meanwhile, high loading of the nucleating agent restricted the ordered arrangement of the molecular chain, leading to low crystallinity. Moreover, during heating, more crystals were formed; as a result, the PLA crystallinity was improved.

**Figure 7:**
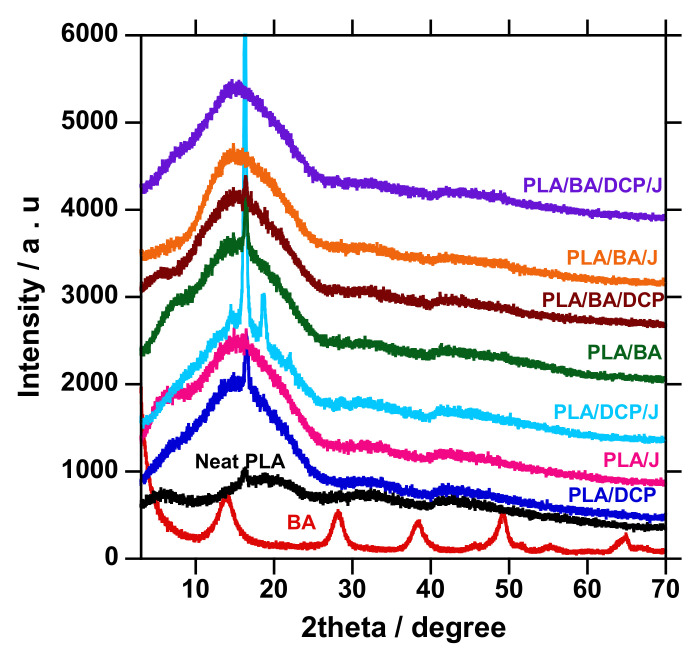
XRD patterns of the neat PLA and composite.

The XRD patterns of neat PLA, BA powder, and composites are shown in Figure 7. The diffraction patterns on the as-received BA powder were observed at 2θ = 13.98°, 28.12°, 38.36°, 49.46°, 55.11°, and 64.60°, attributed to the (20, 120, 031, 200, 002, and 151 crystallographic planes, respectively. In the case of neat PLA, the broad amorphous peak at 16.50° was observed and ascribed to the 200/100 crystallographic plane of PLA crystal, consistent with the features of PLA [38,39,40,41]. Upon the addition of various BA concentrations to PLA, the features of BA at the peak of interest (2¦È = 13.98°) were also recognized, signifying the presence of the filler in the composites. On the other hand, the XRD patterns of all of the composites show an intensive peak around 16.21°, and have slightly moved to a higher angle, suggesting that the crystal size of the composites has decreased due to the interaction and distribution of BA in the PLA matrix [2]. Chain branching contributes to the higher crystallinity of PLA.

Similarly, this tends to reduce the crystal sizes. Therefore, it was important to calculate the crystallite size of the samples, using the Scherrer equation shown below (Equation (3)) as a mathematical expression of the relationship between full width at half maximum FWHM and the crystallite size. The results of the crystalline size for all samples are listed in Table 4.

(3) $$FWHM=\frac{K\gamma }{L\cos\theta }$$

where *FWHM* is the full width at half maximum attained from the instrument, λ is the wavelength of the X-ray that was used for the diffraction, *L* is the crystalline size, *θ* is the peak position (2θ/2) in radians obtained from the instrument, and *K* is a shape factor constant with a value of 0.9 [42]. Neat BA shows a crystalline size of 39.59, and PLA 12.8, with a *T_m_* of 169.11 °C from DSC curves. When a polymer is heated at the minimal *T_m_* and the equilibrium *T_m_* (*T_m_*^0^), the remaining well-ordered structures in the melt will significantly influence the crystallinity [43]. Upon alteration of the PLA structure, the crystal size decreased, and the addition of various BA loading further increased the crystal size with 5 wt.% as the threshold. The small crystalline size is due to the crystal growth of the polymer, which is attained by the extra addition of folded polymer chain segments, meaning that the sample has a lower *T_m_* value [44]. Farid et al. [45] reported a similar observation. Further, the crystal size was not dependent on the BA concentration. In conclusion, we observed that the BA and the resultant branched structure acted as good nucleating agents, and this observation was consistent with the DSC analysis.

### 3.5. HDT

The HDT results of neat PLA and composites are listed in Table 5. The HDT of neat PLA slightly improved from about 0.6 to 1.77 °C after the addition of BA and molecular structure alteration with DCP and Joncryl, which led to the enhancement of the mechanical properties of PLA. In the case of BA’s inclusion in the PLA matrix, the results show an improvement of 1.7 °C; this increase is motivated by the higher degree of BA crystallinity, owing to its nucleating properties. This result is related to the DSC results shown in Figure 6. Upon the molecular structure alteration of PLA by DCP and Joncryl, the HDT increased by 2.0 and 2.7 °C, respectively, due to the chain extension and/or branching.

Further, upon the addition of different BA concentrations, the HDT increased with increasing loading. These results are consistent with the MFR test results reported in Table 2. Overall, the DSC and HDT results show that the incorporation of BA and the alteration of PLA’s molecular structure enhance the distribution of BA particles and the mechanical properties of PLA.

### 3.6. Thermomechanical Properties

**Figure 8:**
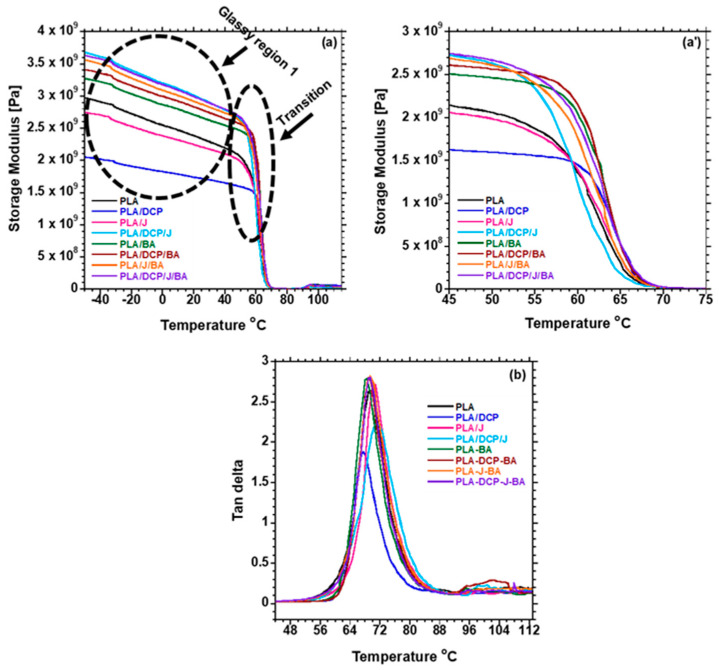
DMA plots of (**a**) storage modulus; (**a′**) storage modulus and (**b**) tan delta curve of neat PLA and composites.

The effects of BA distribution on the thermomechanical properties of PLA were examined. Figure 8a shows the storage modulus (E′) of the samples as a function of temperature. The E′ of the samples is discussed at two different phases: glassy phase, below the *T_g_*, where the polymer chains are highly restricted; and transition phase, at the *T_g_* of PLA (60 °C). The glassy phase shows that neat PLA has a very low E′ compared to the composites. The reinforcing effect of BA in increasing the Eʹ of PLA was noted. However, the addition of DCP further increased the E′ of PLA due to the enhanced distribution of BA and the possibility of branched chains and/or crosslinking, which contributed to the rigidity of the PLA matrix. On the other hand, when Joncryl was added to PLA/BA, it further increased the E′ higher than DCP in PLA/BA, because of the chain extender used to extend the PLA chains.

Furthermore, the concurrent addition of DCP and Joncryl to PLA/BA further increased the E′. The presence of both DCP and Joncryl induces chemical bonding between PLA and BA, as shown in Figure 4; hence, better distribution of BA, as shown in Figure 5. This results in strong interfacial bonding between PLA and BA; hence, the E′ increases when both DCP and Joncryl are added. The increase in E′ can also be attributed to the increase in crystallinity, as shown in Figure 3. With an increase in temperature, the E′ decreased, as expected. Region 2 illustrates the *T_g_* of all of the samples, as listed in Table 6 and shown in Figure 7; the *T_g_* of all of the samples remained almost the same, indicating no effect of BA distribution on the *T_g_*. The *T_g_* of all of the samples examined from the tan delta curve (Figure 7) clearly shows no effect on the *T_g_*, although it was expected that the *T_g_* would move to higher temperatures due to the chain restriction in the presence of BA particles. Overall, it is evident that the storage modulus was dependent on the distribution of BA.

### 3.7. Tensile

**Figure 9:**
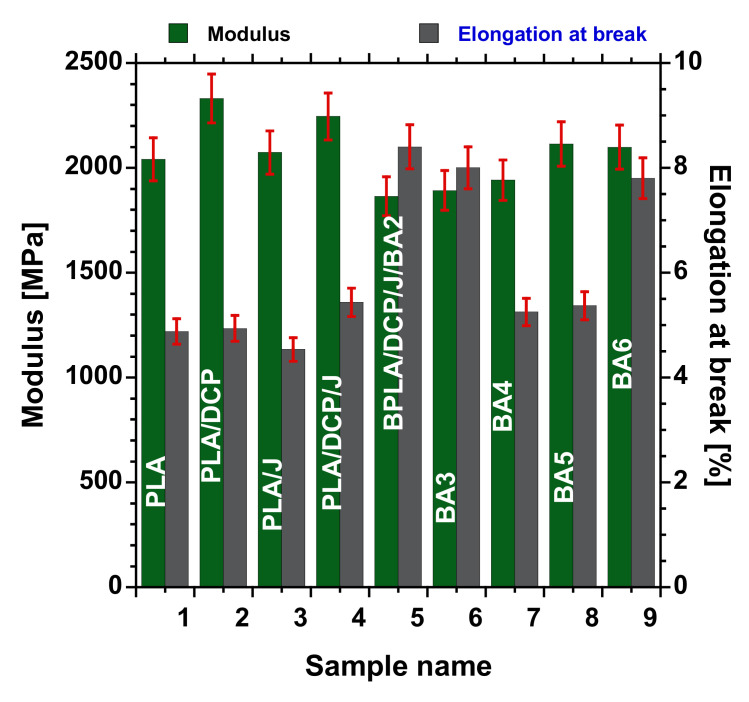
Tensile properties of neat PLA and BA composites with different loadings.

Figure 9 displays the tensile modulus (E′) and elongation at break (ε_ba_) of the neat PLA, PLA/DCP, PLA/J, PLA/DCP/J/, and PLA/DCP/J/BA composites at various concentrations of BA. The neat rigid PLA shows a high E′ of 2040 MPa. Expectedly, PLA exhibits a low ε_ba_ of 4.8%. Upon the structural modification of PLA using DCP and Joncryl individually, the ε_ba_ did not change significantly. However, the E′ of PLA/DCP was higher than that of PLA and PLA/J. The entanglement of crosslinked structures could have caused this increase.

On the other hand, the structural modification of PLA using both DCP and Joncryl concurrently did not change the ε_ba_, while the E′ slightly increased with respect to neat PLA. Upon the addition of 2–4 wt.% BA to the matrix, the E′ slightly decreased, steadily increasing as the BA concentration increased. At the same time, the ε_ba_ increased in low concentrations (2 and 3 wt.%), with a decrease at 4 and 5 wt.% loading. We believe that the good distribution of the filler and improved matrix interaction enhanced the stress transfer of the materials [2]. In this case, the better distribution observed in 3 wt.% loading, as shown in Figure 5, did not lead to the highest enhanced E′ and ε_ba_. Overall, the structural modification of PLA and the incorporation of BA in all of the systems did not significantly affect the E′ and ε_ba_ of PLA.

### 3.8. 3DP

Figure 5e revealed that the BA3 sample showed a fair distribution of BA particles compared to other composites. It is noteworthy that the distribution of the filler particles and the polymer used play key roles in the 3DP process. Due to several extrusions involved in the 3DP process, improved distribution is required in order for heat to be well dissipated in the polymer matrix. Based on that, Supplementary Figure S5 shows the demonstration of the 3D-printed components for neat PLA and BA3 samples. The BA3 sample was chosen from amongst the other composites due to its fair distribution of BA particles in the PLA matrix. The fused deposition modelling (FDM) process based on extrusion technology was used as the technical basis for successfully printing biodegradable PLA/BA composites. A desktop printer (Wanhao Dupilcator i3 plus, Odessa, FL, USA) with a 0.4 mm nozzle was used to produce the 3D-printed specimen. The printing conditions were as follows: nozzle temperature 210 °C; bed temperature 50 °C; print speed of 60 mm/s; 1 perimeter wall; 2 top and bottom layers; and a layer height of 0.2 mm. Supplementary Figure S4 shows different shapes printed by the 3D printer for neat PLA and BA3 samples. A detailed paper concerning this process will follow.

## 4. Conclusions

The present work investigated the effects of strategic modification with DCP and Joncryl on the PLA/BA composites, as well as the influence of BA distribution on the thermomechanical, mechanical, and flow properties. We found that the structural alteration of PLA by Joncryl and DCP had a significant effect on the flow properties and control of degradation. Based on the flow properties and FTIR spectroscopic analysis, the mechanism of stabilization is most likely chain extension. The chain extension leads to the long-chain branched structure in the sample containing Joncryl and DCP. The enhanced distribution of BA particles in the PLA matrix, and the interfacial bonding, were responsible for improving the PLA’s properties. The mechanical properties of the PLA increased. The 3 wt.% BA showed the optimal distribution, and the PLA/DCP/J/BA3 system was chosen for further studies. The produced composite was indeed 3D printable. For future work, it will be interesting to investigate the influence of BA concentration on the PLA/DCP/J/BA system, in order to understand the effects of BA particle distribution on the thermal properties of the resulting system. In conclusion, the structural alteration with DCP and Joncryl successfully improved the BA particle distribution, leading to enhanced thermomechanical and HDT properties of PLA.

## Figures and Tables

**Table 1:** Sample names and compositions.

Sample Code	PLA	DCP	Joncryl	BA (wt.%)
PLA	100	-	-	-
PLA/DCP	99.95	0.05	-	-
PLA/J	99.40	-	0.6	-
PLA/DCP/J	99.35	0.05	0.6	-
PLA/BA	98.00	-	-	2
PLA/DCP/BA	97.95	0.05	-	2
PLA/J/BA	97.40	-	0.6	2
PLA/BA2/DCP/J	97.35	0.05	0.6	2
BA3	96.35	0.05	0.6	3
BA4	95.35	0.05	0.6	4
BA5	94.35	0.05	0.6	5
BA6	93.35	0.05	0.6	6
BA10	89.35	0.05	0.6	10
BA20	79.35	0.05	0.6	20

**Table 2:** The MFR of all samples.

Sample Name	MFR(g/10 min) Using 2.16 kg Load at 190 °C
PLA	15.49
PLA/DCP	10.49
PLA/J	7.71
PLA/DCP/J	7.66
PLA/BA	11.19
PLA/DCP/BA	9.94
PLA/J/BA	5.86
PLA/DCP/J/BA2	6.69
BA3	7.01
BA4	7.78
BA5	8.11
BA6	9.93
BA10	10.50
BA20	11.52

**Table 3:** DSC measurements of neat PLA and composites.

Sample Code	*T_cc_* (°C)	Δ*H_cc_* (J/g)	*X_cc_* (%)	Δ*H_m_* (J/g)	*T_m_* (°C)	*X_m_* (%)	*X_c_* (%)
Neat PLA	110.24 ± 0.8	31.21 ± 1.9	33.31	33.46 ± 0.7	169.11 ± 0.1	35.71	2.40
PLA/DCP	102.72 ± 0.1	26.72 ± 0.1	28.67	31.48 ± 0.1	167.49 ± 0.1	33.80	5.13
PLA/J	108.09 ± 0.1	21.72 ± 0.1	23.32	29.30 ± 0.1	165.78 ± 0.1	31.46	8.14
PLA/DCP/J	-	-	-	28.77 ± 0.1	164.00 ± 0.1	31.08	-
PLA/BA	104.98 ± 0.7	29.47 ± 0.02	32.09	32.56 ± 0.3	167.63 ± 0.7	35.46	3.37
PLA/DCP/BA	105.36 ± 0.3	19.81 ± 0.4	21.58	23.15 ± 0.9	167.28 ± 0.3	25.22	3.64
PLA/J/BA	107.02 ± 0.9	27.20 ± 1.8	29.80	29.35 ± 0.7	166.65 ± 0.5	32.16	2.36
PLA/BA2/DCP/J	105.98 ± 1.0	24.47 ± 0.5	26.98	30.37 ± 0.1	167.14 ± 0.3	33.48	6.50
BA3	106.0 ± 0.6	24.5 ± 0.9	32.0	31.4 ± 0.7	165.51 ± 0.4	33.3	2.8
BA4	102.9 ± 0.9	28.9 ± 0.4	32.6	32.5 ± 0.7	166.60 ± 1.1	34.8	3.8
BA5	105.6 ± 0.3	29.10 ± 0.6	31.2	32.0 ± 0.8	166.72 ± 0.8	36.4	5.0
BA6	106.3 ± 1.2	27.60 ± 0.9	30.4	30.8 ± 0.2	167.10 ± 0.5	35.2	4.8
BA10	108.8 ± 0.9	25.01 ± 0.8	29.87	32.47 ± 0.1	167.98 ± 0.6	38.78	8.91
BA20	110.1 ± 0.6	26.28 ± 1.2	35.34	32.38 ± 0.7	168.53 ± 1.5	43.55	8.21

**Table 4:** Crystal sizes of neat PLA and composites.

Sample Name	2θ	θ	FWHM	Crystal Size (nm)
BA	13.91	6.96	0.038397	36.4
Neat PLA	16.29	8.15	0.119381	11.7
PLA/DCP	16.47	8.24	0.153414	9.1
PLA/J	15.29	7.65	0.213628	6.5
PLA/DCP/J	16.31	8.16	0.008029	174.4
PLA/BA	16.44	8.22	0.117286	12.0
PLA/DCP/BA	16.40	8.20	0.176802	8.0
PLA/J/BA	15.14	7.57	0.205251	6.8
PLA/BA2/DCP/J	14.90	7.45	0.186576	7.5
BA3	16.46	8.23	0.135961	11.2
BA4	16.45	8.22	0.122696	12.4
BA5	16.44	8.22	0.106988	14.3
BA6	14.82	7.41	0.199142	7.6
BA10	16.61	8.30	0.135786	11.2

**Table 5:** HDT values of neat PLA and composites.

Sample Name	HDT (°C)
Neat PLA	51.3 ± 0.21
PLA/DCP	51.9 ± 1.48
PLA/J	54.2 ± 0.40
PLA/DCP/J	53.4 ± 0.29
PLA/BA	53.0 ± 0.06
PLA/DCP/BA	53.3 ± 0.38
PLA/J/BA	54.0 ± 0.15
PLA/BA2/DCP/J	53.1 ± 0.55
BA3	56.8 ± 0.91
BA4	60.2 ± 0.88
BA5	62.2 ± 0.49
BA6	66.7 ± 0.90

**Table 6:** Storage modulus and Tg of neat PLA and composites.

Sample Name	*Tg* °C	Storage Modulus [Pa]
PLA	69.44	2.03 × 10^9^
PLA/DCP	67.36	1.50 × 10^9^
PLA/J	70.13	1.99 × 10^9^
PLA/DCP/J	70.83	2.62 × 10^9^
PLA/BA	68.06	2.38 × 10^9^
PLA/DCP/BA	68.75	2.45 × 10^9^
PLA/J/BA	69.24	2.56 × 10^9^
PLA/BA2/DCP/J	69.44	2.63 × 10^9^
BA3	68.22	2.34 × 10^9^
BA4	69.39	2.27 × 10^9^
BA5	69.84	2.20 × 10^9^
BA6	67.76	2.29 × 10^9^

## Data Availability

Not applicable.

## Supplementary Materials

The following are available online at https://www.mdpi.com/article/10.3390/polym13122019/s1, Figure S1: The mechanism of reaction between PLA and DCP, Figure S2: The mechanism of reaction between PLA and Joncryl, Figure S3: FTIR spectra for: (a) neat PLA, (b) PLA/DCP (c) PLA/J, (d) PLA/DCP/J, (e) PLA/BA, (f) PLA/BA/DCP, (g) PLA/BA, and (h) PLA/BA/DCP/J, Figure S4: FTIR spectra for neat materials of (a) BA, (b) DCP, and (c) Joncryl, Figure S5: The printability of: (a) neat PLA square shape, (b) BA3 square shape, (c) neat PLA CSIR logo, and (d) BA3 CSIR logo.
