# Supplementary material for: Flow Characteristics, Mechanical, Thermal, and Thermomechanical Properties, and 3D Printability of Biodegradable Polylactide Containing Boehmite at Different Loadings
Source: Polymers (Basel). 2021 Jun 21;13(12):2019. doi: 10.3390/polym13122019 (PMC8234614; doi:10.3390/polym13122019)
Supplement: Supplementary file 1 [file polymers-13-02019-s001.zip › polymers-1232460-supplementary.pdf]

polymers

MDPI

*Supplementary Material*

# Flow Characteristics, Mechanical, Thermal, and Thermomechanical Properties, and 3D Printability of Biodegradable Polylactide Containing Boehmite at Different Loadings

**Dimakatso Makwakwa [1,2], Vincent Ojijo [1], Jayita Bandyopadhyay [1] and Suprakas Sinha Ray [1,2,*]**

[1] DST-CSIR National Centre for Nanostructured Materials, Council for Scientific and Industrial Research, Pretoria 0001, South Africa; DMakwakwa@csir.co.za (D.M.); vojijo@csir.co.za (V.O.)
[2] Department of Chemical Sciences, University of Johannesburg, Doornfontein, Johannesburg 2028, South Africa; jbandyopadhyay@csir.co.za
* Correspondence: rsuprakas@csir.co.za

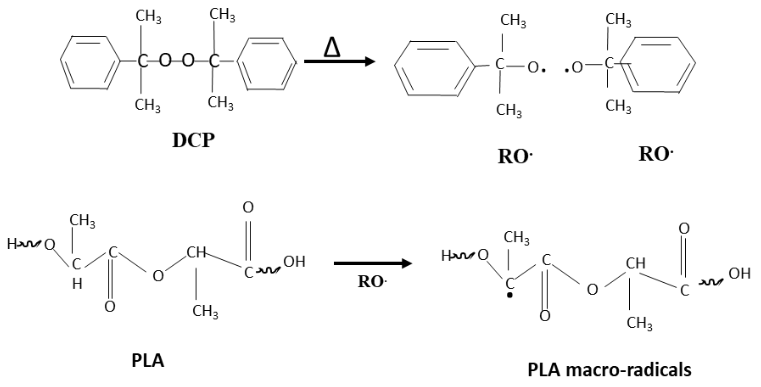


**Figure S1.** The mechanism of reaction between PLA and DCP.

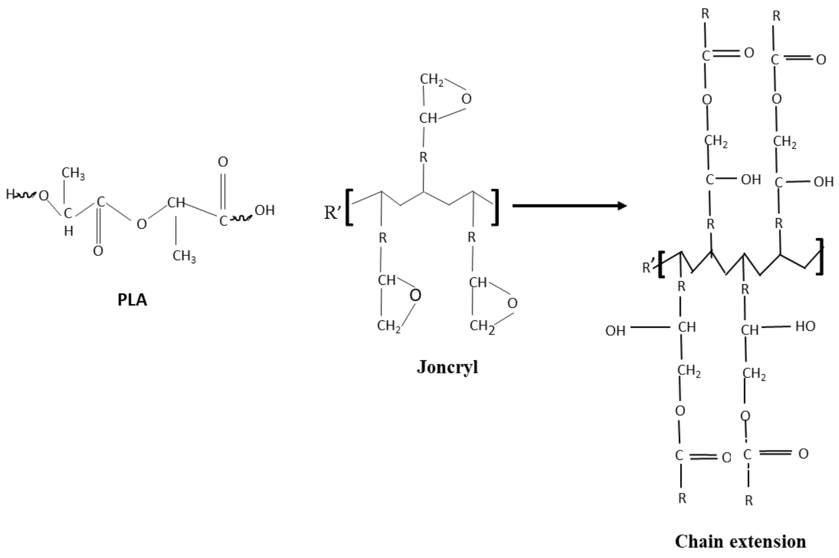


**Figure S2.** The mechanism of reaction between PLA and Joncryl.

**Citation**: Makwakwa, D.; Ojijo, V.; Bandyopadhyay, J.; Ray, S.S. Flow Characteristics, Mechanical, Thermal and Thermo-Mechanical Properties and 3D Printability of Biodegradable Polylactide Containing Boehmite at Different Loadings. *Polymers* 2021, *13*, 2019. https://doi.org/10.3390/polym13122019

Academic Editors: Antonella Patti and Domenico Acierno

Received: 07 May 2021
Accepted: 02 June 2021
Published: 21 June 2021

**Publisher's Note:** MDPI stays neutral with regard to jurisdictional claims in published maps and institutional affiliations.

**Copyright:** © 2021 by the authors. Submitted for possible open access publication under the terms and conditions of the Creative Commons Attribution (CC BY) license (https://creativecommons.org/licenses/by/4.0/).

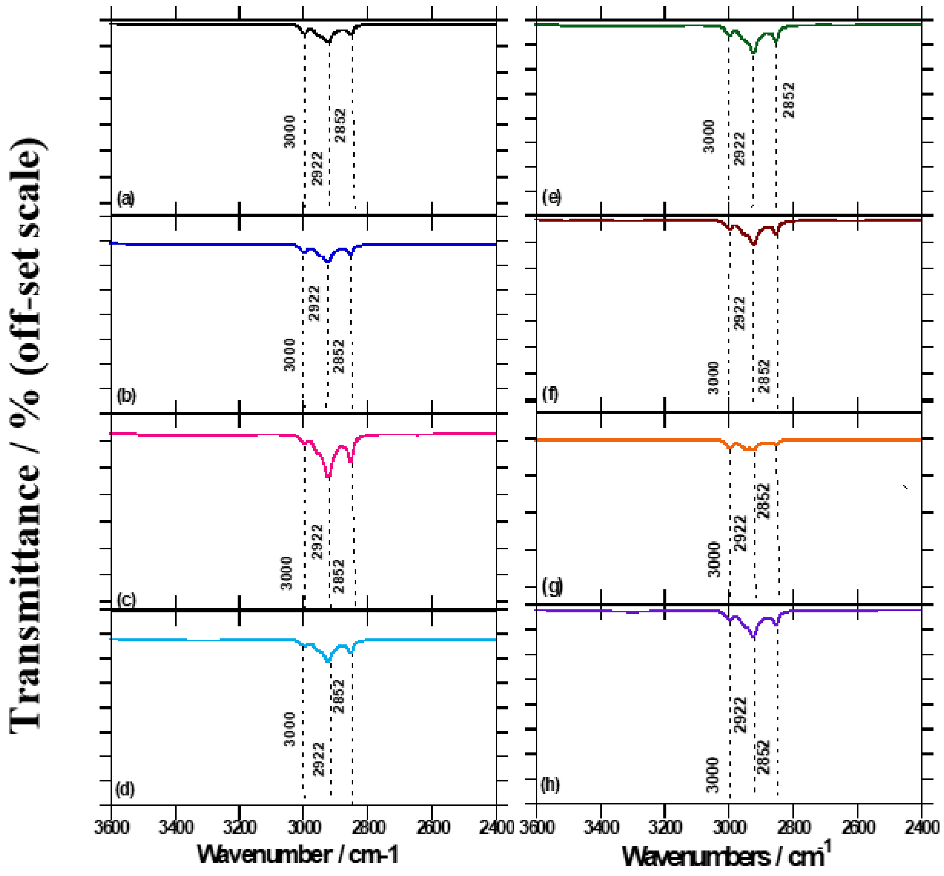


**Figure S3.** FTIR spectra for: (**a**) neat PLA, (**b**) PLA/DCP (**c**) PLA/J, (**d**) PLA/DCP/J, (**e**) PLA/BA, (**f**) PLA/BA/DCP, (**g**) PLA/BA, and (**h**) PLA/BA/DCP/J.

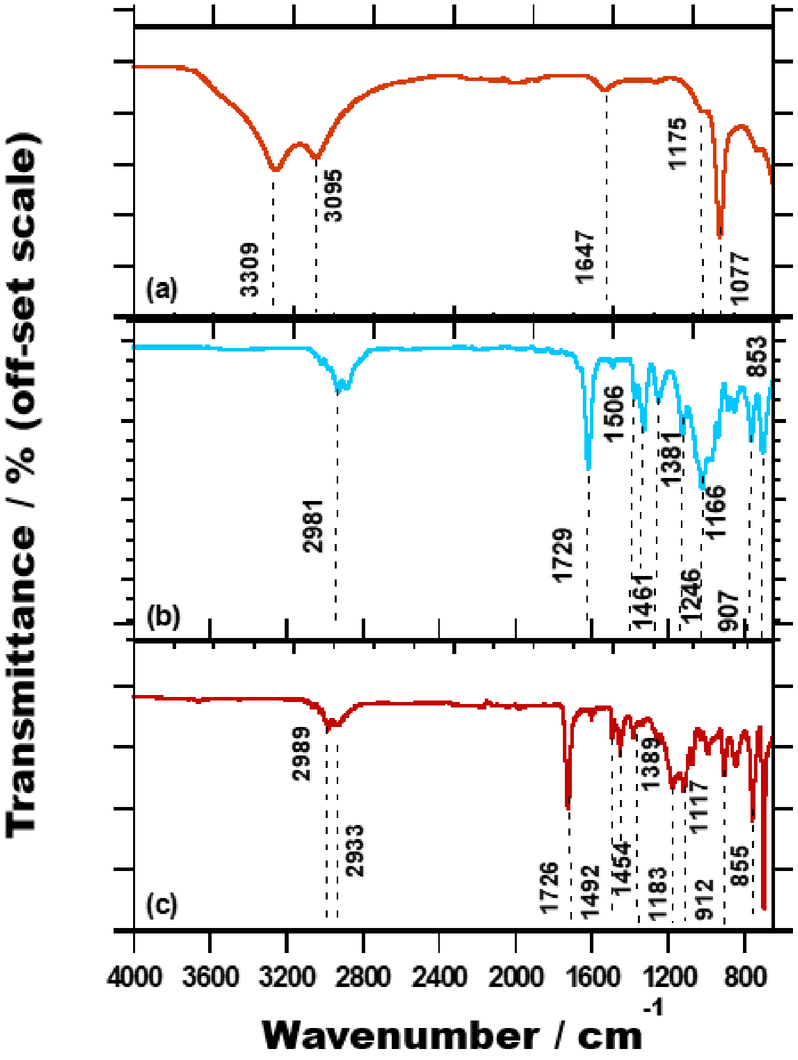


**Figure S4.** FTIR spectra for neat materials of (**a**) BA, (**b**) DCP, and (**c**) Joncryl.

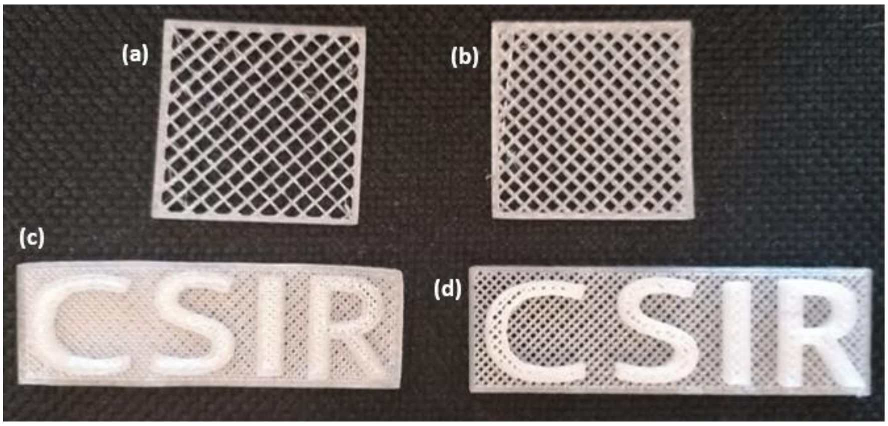


**Figure S5.** The printability of: (**a**) neat PLA square shape, (**b**) BA3 square shape, (**c**) neat PLA CSIR logo, and (**d**) BA3 CSIR logo.
